# Barriers, facilitators and proposed solutions to equitable mental health financing and service delivery for the Lebanese populations and displaced Syrians in Lebanon: Findings from a qualitative study

**DOI:** 10.1371/journal.pgph.0003318

**Published:** 2024-06-28

**Authors:** Rozane El Masri, Sandy Chaar, Joseph Elias, Bassel Meksassi, Rayane Ali, Bayard Roberts, Felicity L. Brown, Michele Kosremelli Asmar, Martin McKee, Rabih El Chammay, Neha S. Singh

**Affiliations:** 1 Research and Development Department, War Child Holland, Beirut, Lebanon; 2 Faculty of Public Health and Policy, London School of Hygiene and Tropical Medicine, London, United Kingdom; 3 Higher Institute of Public Health (ISSP), Saint Joseph University of Beirut, Beirut, Lebanon; 4 Department of Psychiatry, Faculty of Medicine, Saint Joseph University, Beirut, Lebanon; 5 National Mental Health Programme, Ministry of Public Health, Beirut, Lebanon; The Ohio State University, UNITED STATES

## Abstract

Forcibly displaced populations experience an increased burden of mental illness. Scaling up mental health (MH) services places new resource demands on health systems in crises-affected settings and raises questions about how to provide equitable MH services for refugee and host populations. Our study investigates barriers, facilitators, and proposed solutions to MH financing and access for Lebanese populations and Syrian refugees in Lebanon, a protracted crisis setting. We collected qualitative data via 73 interviews and 3 focus group discussions. Participants were purposively selected from: (i) national, United Nations and NGO stakeholders; (ii) frontline MH service providers; (iii) insurance company representatives; (iv) Lebanese and Syrian adults and parents of children aged 12–17 years using MH services. Data were analysed using inductive and deductive approaches. Our results highlight challenges facing Lebanon’s system of financing MH care in the face of ongoing multiple crises, including inequitable coverage, dependence on external humanitarian funds, and risks associated with short-term funding and their impact on sustainability of services. The built environment presents additional challenges to individuals trying to navigate, access and use existing MH services, and the social environment and service provider factors enable or hinder individuals accessing MH care. Registered Syrian refugees have better financial coverage to secondary MH care than Lebanese populations. However, given the economic crisis, both populations are facing similar challenges in paying for and accessing MH care at primary health care (PHC) level. Multiple crises in Lebanon have exacerbated challenges in financing MH care, dependence on external humanitarian funds, and risks and sustainability issues associated with short-term funding. Urgent reforms are needed to Lebanon’s health financing system, working with government and external donors to equitably and efficiently finance and scale up MH care with a focus on PHC, and to reduce inequities in MH service coverage between Lebanese and Syrian refugee populations.

## Introduction

The United Nations High Commissioner for Refugees (UNHCR) estimated that, in 2022, around 110 million persons had been forcibly displaced by war, of which 36.5 million were refugees outside their home country [1]. The global burden of mental illness among those displaced reflects their experiences of conflict, persecution, and human rights violations as well as the unstable environments in which they find themselves [2, 3]. Current guidelines for humanitarian settings recommend an inter-sectoral approach to mental health and psychosocial support (MHPSS) services, including integration of MHPSS considerations into basic services, strengthening community and family support, providing non-specialised mental health (MH) care, and offering specialised services [4]. This approach can be challenging as it requires coordination and prioritisation across different sectors, each with their own financial processes [5, 6]. Moreover, scaling up MH services places new resource demands on already strained health systems in crises-affected settings, including enhanced administration and governance arrangements, additional human resources, upgraded infrastructure, increased access to medicines and strengthened surveillance systems [7–9]. Financing these costs and overcoming access barriers is thus a major concern for countries moving towards universal health coverage, and even more so for countries hosting large numbers of displaced populations. The Inter-Agency Standing Committee (IASC), the main global coordinating mechanism and technical body for MHPSS, therefore recommends creating MHPSS technical working groups at national level to oversee the necessary collaboration and optimise resource utilisation [5, 10].

### The Lebanese context

Lebanon has a long history of armed conflict and political turmoil, and has been impacted by regional wars and other crises. It hosts approximately 1.5 million Syrian refugees, 795,322 of whom are registered with UNHCR [11], with a further 487,000 Palestinian refugees [1]. This refugee population constitutes roughly one-fourth of Lebanon’s total population. The situation of Syrian refugees in Lebanon is dire and there is a lack of official recognition of their status. Since 2015, the Lebanese authorities have implemented restrictive measures, including a work ban, border closures, and stringent residency regulations [12, 13]. This legal and administrative limbo poses practical challenges in accessing education, employment, and healthcare [14]. The situation is further complicated by escalating tensions between Syrian refugees and Lebanese communities, as well as rhetoric from Lebanese politicians attributing unemployment, instability, and diseases to refugees [13, 15].

Since 2019 the country has faced even more economic, political, financial, social and health crises. The progressive devaluation of the currency, rampant inflation, and capital flight were soon joined by the COVID-19 pandemic and, in August 2020, the Beirut Blast and its aftermath. Following the conclusion of previous President’s term in October 2022, the parliament has been unable to elect a new president, adding to the complex political and economic crisis the country has been struggling with for the last four years. These crises have placed additional stress on all sectors of society, especially the under-resourced public infrastructures and services. The currency devaluation of the Lebanese pound (LBP) by 90% since late 2019 has led the World Bank to redesignate the country as Lower Middle income since July 2022 [15]. These recent crises have further increased unemployment, exacerbated poverty, and the availability of services in the country for both host and refugee communities, with implications for MH [16, 17]. A nationally representative survey of Syrian refugees in Lebanon in 2022 found that 90% of families needed support to meet their basic survival needs [16], and 80% of the Lebanese population is estimated to be living below the relative poverty line in March 2023 [18].

### Overview of the Lebanese health system

Universal health coverage is at the heart of the vision for the health system in Lebanon [19]. Yet as of today, the Lebanese health system, a public-private partnership, is nowhere close to achieving this goal. There are multiple sources of funding and channels of delivery depending on which group people belong to, i.e. refugee, migrant or host population, creating many gaps [14]. Lebanon has historically received external aid to support Syrian and Palestinian refugees, but since the economic crisis, the international community is increasingly providing large amounts of humanitarian assistance to vulnerable Lebanese as well, and to prop up public institutions including health care, public education, social assistance, and security. In 2022, UN agencies alone provided $300 million in assistance to or through Lebanon’s public institutions, which is a quarter of Lebanon’s annual public spending [20].

Even though Lebanon has a very high number of health insurance operators, the majority of the population cannot afford to pay for full coverage [21]. Almost 50% of the Lebanese population are enrolled with the National Social Security Fund (NSSF), Lebanon’s social insurance system for employees, or other governmental (i.e. civil servants cooperative and military) schemes [21]. However the NSSF has been recording a deficit for several years. In principle, those outside these schemes are entitled to coverage by the Ministry of Public Health (MoPH) for secondary and tertiary care at both public and private institutions, as a funder of last resort. In practice, the actual coverage provided by public funds is negligible due to the depreciation of the Lebanese currency since the start of the economic crisis. For example, the NSSF can only cover around 10% of health care costs due to NSSF only covering fees in LBP based on lower exchange rates. Consequently, service users have resorted to private insurance companies or paying out-of-pocket, with such payments increasing from 33.1% in 2018 to over 85% in 2022 [22]. Palestinian refugees have health care coverage from the United Nations Relief and Work Agency for Palestinian refugees (UNRWA) [23, 24], while registered Syrian refugees are covered by UNHCR for secondary and tertiary care [14, 25].

The MOPH also provides in-kind support to a national network of primary health care centres (PHCs) across Lebanon that are operated by a mix of local and international non-governmental and faith-based organisations [26, 27]. These PHCs offer consultations with medical specialists at reduced or no cost, as well as medicines for chronic illness and vaccines, which are funded by the MOPH [23]. It is estimated that 68% of PHCs in the MOPH network are owned by NGOs while 80% of hospitals belong to the private sector [28]. The strong presence of the private sector in service delivery has led to a relative oversupply of hospital beds and technology, and while there is an oversupply of physicians, there is a shortage of nurses [29, 30].

The ongoing crises in Lebanon have also heavily impacted the populations’ access to essential medications. Despite spending more than 25% of healthcare expenditure on pharmaceuticals, stocks of drugs have dropped by 50% since the beginning of the economic crisis, leaving more than 70% of the Lebanese population without access to critical medications [22].

### MHPSS system and financing in Lebanon

It is estimated that 90% of individuals needing MH services are left untreated [31]. This treatment gap can be attributed to several factors, including insufficient financial resources, the societal stigma attached to mental illness, a scarcity of qualified MH providers, and misdiagnosis [32, 33]. MHPSS services are delivered through multiple sectors in Lebanon, including health, education, child protection and protection. In 2014 the MOPH launched the National Mental Health Program (NMHP), supported by WHO, UNICEF, and International Medical Corps (IMC). It aims to reform the MH system in Lebanon as a whole, including improvement and scaling-up of primary care-level MH services by implementing the World Health Organization’s Mental Health Gap Action Programme (mhGAP) in PHCs, addressing treatment gaps, reducing stigma, and enhancing the capacity of health professionals [32, 34–36]. As part of the establishment of NMHP, a Lebanese MHPSS Taskforce was also established alongside the establishment of the NMHP, with the mission of ensuring an effective, coordinated response to the MHPSS needs of individuals in Lebanon and aligning services with the national MH strategy [34].

Despite these reforms, Lebanon’s healthcare system, including MH provision, continues to be dominated by the private sector, focused largely on hospitals [37]. To rebalance this, the MOPH and NMHP have partnered with NGOs to expand the network of PHCs, and has been piloting an integrated package of MH care in select PHCs [31, 38, 39]. This integration of MH into PHCs has been challenging due to the fragmentation of the Lebanese health system and the absence of a unified PHC model, although since 2021, the integration of a package of mental services into PHCs is being piloted and is in the process of being costed. However, the dominance of the private sector limits the effectiveness and reach of PHCs.

MHPSS financing arrangements in Lebanon are similar to those in other humanitarian settings where funding priorities are decided based on the organisations leading the response, in this case through the Lebanon Crisis Response plan [17]. The available funds are clearly inadequate to meet the needs [32, 36]. MH accounts for only 5% of the overall health budget of the MOPH and that is mainly for long-term inpatient care in private hospitals [40]. Against a background of limited financial government commitment, the search for alternative sources of funding has been elusive, a situation exacerbated by the political and economic crises. A global review of MH funding allocation observed that international donors rarely aligned funds disbursed for MH with needs [41]. Additionally, more work is needed to understand the challenges associated with the current model of MH service delivery available at the PHCs associated with the NGOs, as these services continue to be piloted and scaled up.

In Lebanon, the last comprehensive study on MH financing was published in 2014 [42], and it only considered the Lebanese population and not Syrian refugees. There have been some studies of MH service provision [36], but again, none so far have looked at MH service provision or access from the perspectives of both the Lebanese population and Syrian refugees, taking account of the impact of the Syrian crisis on Lebanon, or the collective views of MH actors in both the national and humanitarian health systems in Lebanon. Furthermore, it is known that beyond financial considerations and availability of services, a wide variety of factors, including those at the society, family and individual levels influence individual service usage [43]. Accordingly, our study aims to investigate barriers, facilitators, and proposed solutions to MH financing and access for both Lebanese populations and Syrian refugees, with a focus on the MH system in Lebanon. Through this study, we aim to address a critical gap in the existing literature and to provide relevant policy, programmatic and research-relevant recommendations for key MH actors in both national health and humanitarian systems globally as well as in Lebanon.

## Methods

### Conceptual framework

The conceptual framework underpinning our study is that proposed by Ryvicker (2018), applying a behavioural-ecological perspective on healthcare access and navigation [43]. This builds on previous concepts of navigating health care [44], expanding upon their environmental dimensions. Given the challenges with MH funding in Lebanon, we aim to explore the impact on individuals more broadly and to explore how the funding situation interacts with other factors influencing MH service access. Ryvicker’s framework theorises that healthcare navigation is an ecologically informed process not only because of the spatial distribution of health services and resources (including financing), but because of the spatial distribution of individual and environmental factors that influence individual decision-making, behaviour and available resources (including financing) with respect to service use.

### Study setting

This study was conducted in Lebanon, mainly the Beirut and Mount Lebanon (BML) governorates, urban settings that include almost around 50% of the population [45]. This region hosts around 29% of the most deprived groups in the Lebanese population, 22% of registered Syrian refugees, 22% of the poorest Palestinian refugees, 14% of the poorest Palestinian refugees from Syria, and the majority of refugees from countries other than Syria [45, 46].

### Study participants

We conducted 73 interviews and 3 focus group discussions (Table 1). Participants were purposively selected from the following groups between 1 November 2022 and 24 February 2023: (i) national stakeholders including governmental MH authorities, insurance company representatives, UN, donor and NGO staff working in MHPSS policy and management of MH programming; (ii) frontline MH service providers in both public and private sectors; (iii) insurance company representatives; (iv) Lebanese and displaced Syrian parents of children aged 12–17 year using MH services; (v) Lebanese and displaced Syrian adults accessing MH care. We drew our samples from the following sources, using snowball sampling: (i) MHPSS task force members; (ii) government and partner programmes; (ii) community representatives and groups; (iii) MH service providers. We purposively sampled Lebanese and displaced Syrian parents of children aged 12–17 years and adults accessing at least one the following types of care in BML governorates: (i) individual or group psychosocial support at the community level from specialised NGOs providing services at the community level but not as part of the PHC network; (ii) non-specialised mental services for moderate or severe depression, anxiety and/or PTSD at the PHC level; (iii) specialised services for moderate or severe depression, anxiety and/or PTSD at the PHC levels (by a psychiatrist or a psychologist); (iv) care for moderate or severe depression, anxiety and/or PTSD from specialised NGOs providing MH services at the community level but not as part of the PHC network; and (v) care for moderate or severe depression, anxiety and/or PTSD in private facilities. We ensured that we had a balanced gender mix.

**Table 1:** Study participants.

Respondent group	Sample size
Key informant interviews (KIIs)	
National stakeholders	24 including: • 5 UN staff • 2 Insurance representatives • 13 NGO staff • 4 Ministry Public Health stakeholders
Primary healthcare centre (PHC) managers	2
Mental health (MH) service providers from PHCs and community-based NGOs	6 including: • 2 social workers • 3 psychologists • 1 psychiatrist
Lebanese parents of children aged 12–17 years	5 including: • 1 male • 4 females
Displaced Syrian parents of children aged 12–17 years	5 including: • 2 males • 3 females
Lebanese adults receiving MH care	16 including: • 3 males • 13 females
Displaced Syrian adults receiving MH care	15 including: • 3 males • 11 females • 1 trans woman
**Total KIIs**	**73**
Focus group discussions (FGD)	
Displaced Syrian service users	1 comprising of 4 females
Lebanese service users	2 • 1 FGD with 3 females • 1 FGD with 7 males
**Total FGDs**	**3**

Table 1 provides an overview of the number of interviews and focus groups we conducted with each respondent group.

### Data collection tools and processes

Key informant interviews were carried out by a team of Lebanese researchers (REM, JE, BM, RA, SC) working in a Lebanon-based International NGO working in child protection, education, and MHPSS, as well as one academic from a UK university (NSS). All had prior experience in conducting qualitative research and received five days of training on MH access and financing, qualitative research and research ethics for this project.

**Fig 1:**
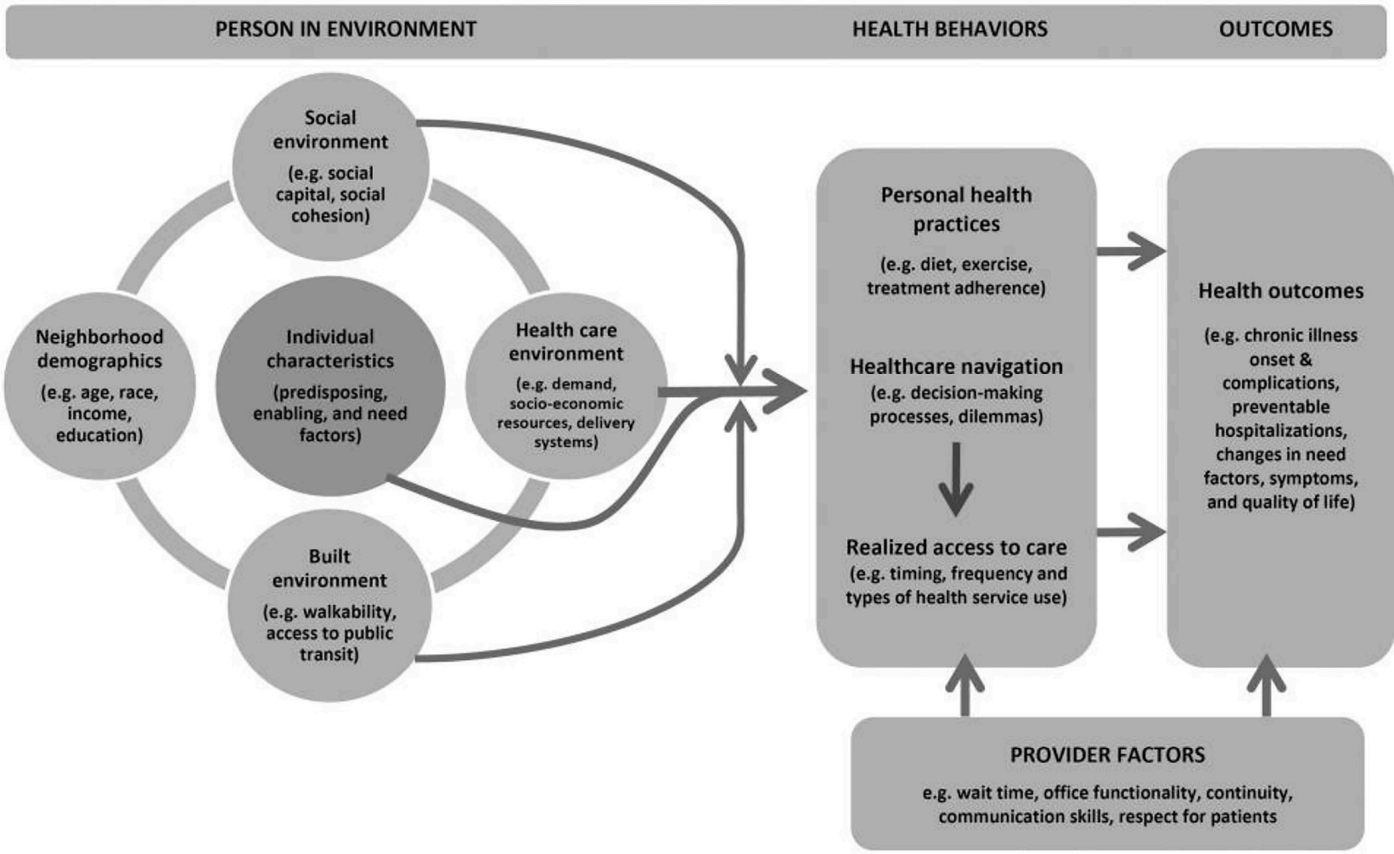
Conceptual framework for examining healthcare access, financing and navigation [43].

Interview topic guides were informed by the Ryvicker conceptual framework (Fig 1), and designed to answer the research question, which concerned barriers and facilitators to accessing equitable MH services for Syrians and Lebanese and were adapted to the situations of different groups. Interviews with service users and caregivers adopted a narrative approach, in which the participant is asked to tell their story of navigating and accessing the services, with subsequent probes as needed to explore health care seeking and financing in relation to MH services. Narrative interviewing gives the participant control over how they share their stories, allowing a relaxed and unstructured approach that can elicit depth and, at times, more accurate information than obtained in a structured interview [47, 48]. This contributes to dismantling power hierarchies linking the researcher and participant [49, 50]. With national stakeholders and service providers, a semi-structured format was used. All topic guides were tested and piloted with study team members with experience in MHPSS programmes with Lebanese and displaced Syrians and refined accordingly, and with service users.

Interviews were conducted by one researcher, primarily in person at the healthcare facility that the service user/caregiver parent usually attends, their houses, or the stakeholder’s office. Participants were also offered the option of remote interviews, some conducted over Zoom. Interviews were conducted in English or Arabic, depending on the participants’ preference. All interviews were audio-recorded, and lasted between 45 and 90 minutes.

### Data analysis

The interviews were transcribed verbatim in English and from Arabic to English. Quality checks were conducted for all the transcripts by REM. Data were coded using an inductive and deductive approach based on the framework method [47], with a coding framework developed including themes based on the Ryvicker conceptual framework (Fig 1) on healthcare access and navigation (2018). Dedoose software was used for coding the data. Six Authors (NSS, REM, JE, BM, RA, SC) coded the data in pairs and used collaborative coding approach [48] to analyse, define and refine the codes where they held group meetings for that purpose. During these meetings, each pairs’ coding was checked with the team of authors and key themes of each transcripts were identified and discussed collaboratively. Data saturation was reached, with later transcripts not generating new codes.

We invited all study participants to feedback sessions to enable them to reflect on emerging findings and provide feedback. Involving target populations in the data interpretation and synthesis stage is recommended when implementing a decolonial and participatory approach to research in forced displacement settings [49]. Group meetings for national stakeholders, facility managers and service providers took place over Zoom or MS Teams, with findings shared via email for their feedback if they were unable to join the group meetings. Feedback sessions with service users were held individually via Zoom or a WhatsApp call. We also used short video recordings followed by discussion on WhatsApp with participants as needed, as this method has been noted as highly feasible in Lebanon [50].

### Ethics approval and consent to participate

Ethical approval was obtained from Saint Joseph University in Beirut (ref: USJ-2020-224, 19/01/2021), and the London School of Hygiene and Tropical Medicine in London (ref: 22766, 13/01/2021). Participants were provided with a Participant Information Sheet detailing the aim and scope of the study, and written informed consent was then obtained from participants prior to conducting interviews and focus groups. All identifying information in the transcripts was anonymised and numerical codes were assigned to each transcript. All participants provided written informed consent to take part in the research, conducted by researchers prior to commencement.

## Results

We present the enablers, barriers and proposed solutions to MH financing and services for Syrian refugees and Lebanese populations in Lebanon. We found that the multiple crises that Lebanon has been facing–economic, political, COVID-19, and the Beirut blast–all influence how MH services are financed, delivered to and accessed by Syrian refugees and the Lebanese population. We organise our findings according to seven themes that mostly follow our conceptual framework: (1) healthcare environment; (2) built environment; (3) healthcare navigation; (4) provider factors; (5) social environment; (6) gender norms; and (7) individual characteristics. Overviews of the barriers and enablers are presented in Table 2, and proposed solutions from study participants are presented in Table 3.

**Table 2:** Overview of barriers and facilitators to financing and access of mental health services for Syrian and Lebanese populations in Lebanon.

Theme	Barriers	Facilitators
Healthcare environment	Lack of government funding for mental health (MH) services, leading to reliance on external aid	
	The high cost of MH services in secondary and tertiary care including medication	Re-focusing MH care at PHC level and making MH care and medication free or low-cost is enabling better access
	Poor and under-resourced MH departments in public hospitals and primary healthcare centres (PHCs)	Commitment of MOPH and NMHP to shift focus of Lebanon’s MH system from secondary and tertiary care to community-based services including primary care level services; and to train PHCs and NGOs
	The shift towards free or low-cost services creates increased demand on PHCs and NGOs, leading to long waiting lists and limited capacity	People who can borrow money from family and friends are better able to access MH care and related medications
	Financial constraints can result in non-adherence to treatment, such as medication, and self-medication, causing further harm to individuals	MOPH is covering costs for Lebanese citizens who are not covered by social security–however, it is hard to navigate getting this coverage
	Shortage of MH service providers and “brain drain”	After the Beirut blast, Additional MH services were provided and made available in affected areas around Beirut.
	Influx of refugees increased the demand for MH services, straining the available resources	Multiple crises in Lebanon have changed the attitude of people toward accepting and seeking MH support, in particular amongst men
	Better financial coverage for Syrians registered with UNHCR versus Lebanese for in-patient hospitalisation; and insurance companies only accepting USD during the economic crisis, which limits Lebanese populations; service users’ access to insurance	Syrians with active UNHCR registrations have financial coverage for secondary and tertiary level MH services through UNHCR/NGOs
	Severe medication shortages in the Lebanese market and challenges in finding substitutes due to the economic crisis	
Built Environment	High cost of transportation and insufficient transportation reimbursement for service users from NGOs	NGO-led PHCs supporting access in terms of covering transportation cost of service users
	Internet and electricity challenges for remote services	Facilities’ flexibility in scheduling appointments and mode of delivery (remote, over phone, in person)
	Lack of equitable geographic coverage and dedicated rooms for MH sessions	Public MH services are starting to be embedded within a system of PHCs available throughout the country
Healthcare navigation	Lack of standardisation of the process of healthcare access for patients at the PHC level	
	Patients’ lack of knowledge and confusion around the process	
Provider factors	Poor quality consultations and lack of interest beyond providing a medical diagnosis	Service providers’ building rapport with service users and demonstrating care and continuous support for them
	High burnout among service providers	Service providers respecting patients’ confidentiality
		Service providers setting expectations with service user around MH services
		Service providers’ demonstrating sensitivity to diverse sexual orientations and gender identities
		Service providers’ working to normalise service users’ feelings towards accepting MH services and alleviating related stigma
Social environment	Social environment exacerbating MH conditions	Support and encouragement of family and friends
	Stigma and societal attitudes	The importance of familial and partners’ support in seeking and adhering to MH care
	PHC’s operating mainly during weekdays and during times that are not suitable for working men. Higher dropouts in MH services are recorded for men than women.	After the several crises especially the economic crisis and the blast crisis, Men got more at ease with seeking MH than before. (Also in impact of crises)
Gender norms	Gender roles and economic control play a role in who determines if money can be allocated to paying for MH services. Often, gender roles and patriarchal systems affect women who are denied money for MH care from their husbands or families.	Compared to before the Beirut Blast and economic crisis, more men are now accepting of and accessing MH care. This is in part due to reduction in social stigma relating to MH, as well as increased awareness campaigns related to MH and related services.
	Service providers not able to take into consideration service users’ preference about the gender of the MH provider, due to the lack of male service providers.	
	Women find it challenging to juggle work and childcare responsibilities and find the time to access MH services, also because of fear of judgement by their partners.	
Individual characteristics	Cultural factors leading to misconceptions about medications and MH services	Observable positive impacts of MH services can encourage individuals to recognise its value and seek help
	Harmful coping mechanisms and alternative treatment options	
	Lack of knowledge about the importance of therapy and concerns about self-confidence hinder help-seeking behaviours	

**Table 3:** Proposed solutions from study participants to improve mental health care financing and access for Syrians and Lebanese populations in Lebanon.

	Proposed solutions
**National leadership and resources for mental health (MH) care**	While there is commitment to MH at the MOPH level, there is an urgent need for the government to raise funds for the National MH Programme (NMHP) and to fully implement the National MH Strategy.
	MOPH should **explore innovative financing mechanisms** (to supplement humanitarian funding) to make critical improvements in coverage for MH (including services and medication) at the primary healthcare (PHC) level. This could include channelling humanitarian funding into the national system, rather than into a parallel system.
	Building on the existing order for psychologists, MOPH should expand its regulation and standardisation of additional MH professionals by creating and implementing a national certification process, e.g. for MH nurses and community health workers. This work should also be undertaken for social workers under the auspices of the Ministry of Social Affairs.
	Recognising that there is already a dedicated workstream on this in the MHPSS taskforce working on trying to harmonise fees, relevant stakeholders should **work on allocating and standardising specific payments and allocation of funds for NGOs and MH specialists on a national level**, to avoid competition over resources between the different NGOs. This also leads to inequity in pay between staff employed by MOPH and NGOs.
	MOPH and NMHP should **continue investing in and building the capacity of PHCs** for them to expand and sustain MH services.
**Improving access to and integration of mental services in PHCs**	Building on the work being done to implement a national model of MH care MOPH and NMHP should work in strong collaboration with all ministries and relevant NGOs to publicise the recently published list of free or affordable MH services (https://mhis.nmhp-lb.com/#/guest/home) with all populations.
	Have **dedicated rooms for MH services in PHCs**
	Consider offering **group therapy sessions**, in addition to individual sessions.
	Have a standardised unit cost for MH services at PHCs to be able to secure more funds through integrated packages of health care
	**Task-sharing of MH services to non-specialist health care providers** at the PHCs will help decrease the current over-reliance on MH specialists
	Conduct **widespread campaigns, co-created with communities, that go beyond specific MH conditions** such as suicide and delve into broader aspects of MH, including various disorders and symptoms
**Funding**	Working with relevant ministries and building on recommendations in the draft MH Strategy 2023–2030 [38], MOPH should **formulate a comprehensive policy framework for health financing guaranteeing social health protection to all.** Coordinated action among the different actors who currently finance MH and provide social health protection entitlements is needed to move further towards equitable access to MH services. This means: • aiming towards mandatory, universal coverage, • a comprehensive benefit package; and • complimentary contributions from workers and employers based on the ability to pay as well as tax-financed contributions. In parallel, a dialogue is needed on concrete ways to reduce current fragmentation of the system, in particular regarding the purchasing function which damages schemes’ ability to negotiate with providers.
	Build on commitments from MOPH to implement combining social health insurance funds in the National Social Security Fund (NSSF) and MoPH funding (from general taxation) in one single-payer pool, in order to maximise efficiency (by reducing administration costs) and increase the scope for cross-subsidies to vulnerable populations
	**Strengthen implementation of the MOPH and NMHP’s commitments towards strategic health financing mechanisms with a focus on PHC** by: • Progressively aligning purchasing methods, at least among public purchasers • Moving away from passive fee-for-service mechanisms for secondary and tertiary care and including a strong primary care component which should play the role of gate keeper. • Unifying tariffs • Pooling procurement of medicines In parallel, **contain costs in the health system** by: • introducing incentives or obligations for patients to seek treatment in PHCs. • Having main public purchasers (especially NSSF, MOPH and UNHCR) share their learnings and good practices in order to reinforce their negotiation power and purchasing techniques
	Reduce fragmentation at all levels. Specifically: (1) **Strengthen NSSF** as the institution with the legal mandate to provide social health protection to the population, through the gradual integration of smaller schemes and risk pools; and (2) **Foster a national consensus on how to cover the population with no or little contributory capacity in an equitable way** on a similar basis as the coverage offered by the NSSF rather than only an “insurer of last resort” mechanism as currently implemented by MOPH due to the important coverage gaps of NSSF.
	**NSSF should review the current practice of denying benefits to non-Lebanese who are working and paying taxes**, which is not in line with the principle of equality of treatment.
	The MOPH should **explore new and strengthen existing partnerships with the private sector**, as they could contribute funding to support the national health system and public services including for MH. Any partnerships should be underpinned by a clear framework and agreements to avoid ethical issues and conflicts of interest.

### Healthcare environment

#### Health financing

Participants highlighted several challenges to promoting equity, effectiveness, and efficiency of the health financing system, each hindering delivery of MH services and increasing dependence on external aid. Participants acknowledged how the fragmentation of the health financing system, with different ministries and institutions making decisions on health expenditure and implementing social health protection schemes (pooling and purchasing). Their overlapping mandates hinder effective operations, as reflected in the numerous social health protection pools covering different population groups while leaving a large share of the population uncovered. Some participants saw a lack of political commitment to reform, with one participant from government noting the *“little interest among politicians to allocate money for primary health care in general and in particular [to mental health]”* (male Lebanese MOPH stakeholder).

It was also widely recognised that, especially since start of the economic crisis, gaps in the coverage of social health insurance funds–namely MOPH and NSSF–have worsened due to the drastic currency devaluation. Importantly, private health insurance schemes do not explicitly cover MH services. Furthermore, while public funding mechanisms and private insurance cover the Lebanese population, they exclude Syrian refugees. However, since the economic crisis, some participants perceived that hospitals are less willing to admit Lebanese patients, as they are reliant on reimbursement by the MoPH or NSSF and this funding is often very limited or unavailable. Consequently, they *“prefer to take… private patients or those covered by UNRWA or UNHCR that they would be paid in USD”* (female Lebanese UN staff) which reportedly further exacerbated the gap in financial coverage between Syrians and Lebanese populations. One Lebanese service user highlighted how inpatient MH care was unaffordable: *“They [MOPH] should pay at least 80%*. *Because no one can afford the whole cost*. *And I want to get into rehab*, *so I discovered something*, *that the ministry doesn’t cover anything anymore these days*. *And I can’t afford this much”* (female Lebanese MH service user). In contrast, Syrian refugees registered with UNHCR have 90% of the cost of secondary and tertiary MH care covered, and pay the same subsidised or no consultation fees when accessing MH services via NGOs or PHCs. Challenges with the fragmented funding arrangements were highlighted by a female Lebanese UN programme manager: “…Now the access to [health] care is more favouring for the Syrian refugee population versus Lebanese because they [Syrian refugees] have a coverage of 100% or 90% coverage from the UNHCR and MH is considered in their SOPs [Standard Operating Procedures] to be fully covered… I think 10% of the [Lebanese] population has private insurance … they can maybe access private facilities, the other [Lebanese] population… are [needing to be covered] at the last resort by the Ministry of Public Health.”

Despite these challenges, participants were encouraged by how the MOPH and NMHP are starting to integrate standardized packages of MH services into PHCs which serve all populations and are *“pushing and highlighting the importance of it”* (female Lebanese NGO staff). However, it was largely acknowledged that progress on scaling up MH services within and across Lebanon’s PHC network has been *“a challenge on the ground”* due to lack of resources and PHCs struggling to meet demand for the services they provide: *“So because they [PHCs] are very overwhelmed*, *there isn’t enough staffing on the PHC level*. *So*, *it’s [MH] not one of their priorities*. *And it’s sometimes a challenge to ensure their commitment”* (female Lebanese NGO staff).

Lack of resources means that service providers are *“mainly relying on humanitarian funds… which are now currently decreasing*, *day by day”* (Female Lebanese UN staff). This creates a short-term project-based culture. One participant noted: *“But their [MOPH] funds are limited*. *It’s mostly international funds and NGO funds who are running these [MH] services”* (female Lebanese NGO staff).

Participants also noted how the lack of MOPH funding for hospital level MH services impacted its availability *“because very few hospitals they have psychiatric care”* (female Lebanese NGO staff). Even when psychiatric care was available in hospitals, it is often very under-resourced and unable to meet demand.

#### Populations under increasing financial pressure

This increased cost of private MH services and the effects of crises have forced many Lebanese service users to seek free or subsidised services at facilities that historically served Syrian refugees or low-income Lebanese: *“Yes*, *all [MH services in] the private sector become very expensive*, *so there are more Lebanese that are benefiting from services through PHCs*, *either health services or MH services*.*”* ((female Lebanese NGO staff). The increasing number of Lebanese people seeking primary health care level MH services has imposed more pressure on PHCs and NGOs at a time when they are already facing shortages of staff, medications, fuel, and electricity. Consequently, patients face long waits for treatment and some face catastrophic spending on private MH care: *“I honestly was looking for an NGO [including PHCs]*, *because they told me others are very expensive if I wanted to go to a private clinic*. *The range varied between $20–100*. *And I don’t know anyone who is willing to pay $100 for a session*. *So*, *one can have second thoughts [to access MH care] because of the high cost”* (male Lebanese service user). Another female Lebanese service user explained: *“I just told you in the NGO I know that there is a very long waiting list*, *and there are a lot of people waiting*, *including my mother*, *where I included her name a long time ago*, *around a year ago*, *and her name hasn’t come out yet*, *because I know there are only 2 psychologists in this NGO*.*”*

Participants who could access NGO services saw them positively. As a Lebanese service user explained: *“Thankfully they [NGOs] are providing everything to the people who have MH issues*. *We pay for transportation*, *but the other things*, *like the doctor’s fees and the tests*, *are either free of charge or cheap”* (female Lebanese service user).

### Delivery systems

Participants described how MH services provided by NGOs under the auspices of the MOPH were unable to meet demand. A PHC manager noted the challenges posed by the large influx of Syrian refugees since 2011: *"They [Syrian refugees] started coming in bigger number*. *[…] We were in 2011*, *monthly 500 people [accessing services at the PHC]*, *now we are around 11 or 12 thousand monthly”* (female Lebanese PHC manager). Lebanese individuals have increased needs since the Beirut Blast, further lengthening waiting lists at PHCs: *"The Syrians at the beginning they were huge in number*. *Our numbers are shifted now*. *We were mostly Syrians and very little*, *very few Lebanese*. *[Now] We are almost equal and sometimes Lebanese*… *more than the Syrians”* (female Lebanese PHC manager). Moreover, the absence of dedicated rooms for MH sessions in healthcare facilities further impeded access, with participants expressing concern about privacy and stigma.

Participants reported acute shortages of medications, including psychotropics. This is partly due to the devaluation of the Lebanese currency, as well problems with distribution and storage of the medications due to lack of electricity and fuel. As one Lebanese service user noted: *“…my medicines are not available*, *and I can’t have them because of their high prices… For example*, *I am without my meds for 27 days”* (female Lebanese service user). Another female Lebanese MH service user said:”*And the medications*, *most importantly*. *Meaning when I will find the medication expensive*, *I might be forced to stop it*.*”* A female Syrian service user mentioned reducing medication doses without consulting health providers because of the cost of procuring medication from Syria that was unavailable when she tried to buy it in Lebanon: *“My brother sent me some medications from Syria*, *but the medicine there is very expensive*, *and he didn’t have a lot of money*. *Since there are no medications*, *I started to adapt*. *Instead of having two tablets per day*, *I started taking one*. *Then I started taking only half a pill*. *Then*, *a pill every 2–3 days*.*”*

Access was also hindered by limited information on services: *“Of course as long as there is a trusted site that has this information regarding the existence of these services*, *in which areas*, *for what purpose*, *then you would know better how to make the decision*, *and not to call to find a therapist*, *but perhaps there would be more information about the specialty*. *This helps in making the decision more efficiently*, *when we know the specialty of the doctor*, *then we would know what we want to treat specifically*. *If we want to compare therapies*, *we might think that we are going to see a general practitioner”* (female Lebanese service user).

There were particular challenges related to the shortage of health workers, with many psychologists and psychiatrists moving overseas. One female Lebanese UN staff member noted that this is: *“really now a major problem in the country*… *I think we used to have like 70 active psychiatrists*, *now we don’t have 30*. *These 30 are mostly taken by the work that’s taking place in NGOs [who run PHCs] and you barely have*, *you know*, *a psychiatrist that has a bit of time to provide services [in these PHCs] or to provide training—and they have their private clinics*.*”*

This shortage overburdened existing staff: *"everyone is overloaded*… *Each social worker*, *I would say here is following up on an average of 300 active patients*.*"* (female Lebanese NGO staff). It also restricted choice: *“And most of the therapists are women*. *I asked to see a male therapist*, *but there wasn’t any”* (male Lebanese service user).

There was a wide gap between what MH cadres can earn working for international NGOs compared with the national health system. Recognising that the MHPSS taskforce has a workstream trying to harmonise fees, participants recommended that work should also be done by relevant stakeholders on allocating and standardising specific payments and allocation of funds for NGOs and MH specialists nationally, to avoid competition over resources among NGOs.

### Built environment

Even when service users can obtain free or subsidised MH services in the public sector, the increased cost of transportation poses a significant barrier: *“I had financial problems here in Beirut*, *where I couldn’t afford to always come and go to the centre [providing MH services]*. *The [MH] services here are almost free*, *as they only charge 15*,*000LL for the session*. *But I couldn’t attend [them]”* (female Syrian service user). Another male Lebanese service user elaborated: *“Regarding the services*, *they asked me if I was able to go to the centre*. *I told them I couldn’t*. *The main reason was because the location of the centre was far away*, *and I didn’t have any means of transportation*, *and financially*. *Gas costs a lot*.*”*

Both Syrian and Lebanese service users, especially those residing in distant towns or villages, grappled with geographical barriers and transportation fees, with MH services concentrated in Beirut. One male Lebanese NGO staff member explained that: *“…some people in some areas need maybe to change maybe 3 buses maybe to reach*, *they are coming from a very far area*, *and walk maybe more time… so that they can save some money so that they can come*.*”* This was also described by participants from underrepresented areas: *“You can’t find [MH] services everywhere*, *you can find most of them in Beirut if you want*, *so the person living far away won’t find this service distributed all over Lebanon*. *So if this service was found on a certain platform or website*, *that shows the specialty of every doctor and the location of every doctor and the prices*, *then the person would be better capable of making a more appropriate decision for themselves”* (female Lebanese service user).

These problems were especially severe for elderly people and individuals with disabilities accessing MH care: *“Regarding elderly*, *they can’t go out of the house because of the lack of electricity [needed for elevators] and such”* (female Lebanese PHC manager). This facility manager noted how this could be eased if PHCs and NGOs providing MH services to “*talk to the notary and see how many geriatrics we have in a certain area and provide a team to perform home visits for them*.*”*

### Healthcare navigation

Service users reported multiple, often ad hoc pathways to finding and accessing mental health services, including acquaintances, extended family members, NGOs and PHCs, as well as the UNHCR for Syrian refugees. Yet several participants also reported challenges, as a Lebanese father of a child accessing MH services noted: *“As a matter of fact I wanted to seek help before*, *…but I didn’t know where to go*.*”*

Participants noted the importance of social networks: *“Yes*, *I remembered something you asked me about*. *You asked me how I was able to access therapy*. *I know a lot of people*. *Because of that*, *I was able to reach [NGO providing MH Hotline services]*, *and eventually to [NGO providing current services he is receiving] clinic*. *It would’ve been super hard for me to reach the clinic if I hadn’t known these people”* (female Lebanese service user).

The flexibility of scheduling and service delivery was also identified as a key facilitator of access although unavailability of internet access and electricity posed a problem: “The remote areas *in Lebanon*… *even though the NMHP is working on a programme*, *which I was a part of*. *It’s called Step by Step*. *These things help us reach remote areas*. *But you need internet to benefit from this*. *There are some people living in rural areas who don’t have access to the internet*, *even though they are few in numbers*, *as I believe the majority of people have internet access now*. *Or they have a slow connection*. *That would bring us back to the problem*. *I think the people of Bekaa*, *the far north*, *the south*, *the areas away from the cities need such services*. *We need more awareness sessions and outreach in order to ameliorate this challenge”* (female Lebanese NGO staff).

Participants argued that more convenient appointment times and innovative service delivery, such as in-person and virtual sessions, would make it easier for them, especially those in work. Remote modalities of service delivery also made reaching people with disabilities easier: *“With the remote modality*, *it’s*, *it’s much easier*, *much easier now [to reach people with disabilities]*. *We can provide remote consultations for people who have access problems or mobility problems*.*”* (female Lebanese NGO manager). Many employers refused their staff time to attend services or recuperate: *“I told my boss that I wanted to take a week off*, *so I can rest*. *He refused*. *That’s when I took the 5 pills and a beer”* (male Syrian service user). Additionally, some participants, such as a Lebanese schoolteacher, expressed frustration at the unavailability of therapy sessions at convenient times.

Participants also recognised how a *"major gap is the referral from the primary level to secondary level*, *to a specialized psychiatrist*. *This remains you know*, *a major problem… after primary [mental] health care*, *you know the conditions that should be dealt with at the higher… more specialised level”* (male Lebanese MOPH stakeholder).

### Service provider factors

Participants saw service providers’ attitudes as both a barrier and facilitator to access. For instance, some perceived providers as only focused on making a diagnosis: *“That’s why I wasn’t comfortable with the first psychiatrist*. *She only needed a diagnosis*.*” (female Lebanese service user)*. *Additionally*, *lack of attendance from service provider was exemplified with them missing their appointment with the service user*: *“And sometimes the specialist won’t attend*, *which is bothersome for the patient*, *as they are paying transportation to be there”* (female Lebanese PHC manager).

Participants also felt upset by a lack of compassion and professionalism by providers: “*She [MH service provider] made me feel really offended*. *I did not like her approach at all*. *I was kind of I felt like I was repeating the same information*. *There wasn’t any compassion*. *There wasn’t any really useful information*, *anything that I didn’t know myself one time*, *it was only like two or three sessions*. *One of the times she had told me at the end ‘how much you fear to be like your mother’*. *To me [this was] totally inappropriate”* (female Lebanese service user).

Many expressed concern about the pressures facing providers: One service provider said that: *“The burnout between employees is faster now*. *When I was an intern*, *before the conflict*, *I didn’t see that much tiredness among service providers”* (female NGO staff member), with a service user noting: *“like they [MH service providers] need therapy as well I’m sure*, *you know*!*”* (male Lebanese service user).

At the same time, we found that service providers who build rapport and display empathy enabled and enhanced service users’ access. One Syrian service user said: *“It was great*. *She [service provider] promised me to speak with the centre and to issue a medical document for me*, *and to find a way for me to contact the UN*. *She took my issue more seriously*, *and she gave me hope*. *She made me feel that someone is standing next to me*. *This made me feel better”* (male Syrian service user). Respecting confidentiality was another important quality in a service provider, as noted by a Lebanese service user: *“They were friendly with me*. *And they were very confidential*. *For example*, *my mother called them to ask about me*, *without me knowing*, *they didn’t give her any information*. *And they check up on me”* (female Lebanese service user).

Service users perceived NGO social workers, a key MHPSS cadre, as supportive, assisting them to navigate the system and providing a patient-centric approach, considering their preferences in scheduling appointments and determining treatment modalities. One female Lebanese service user noted: *“The social worker there helps me as well*, *she comforts me as I told you*, *as she sometimes helps me to organize the time and such*.*”*

Participants also agreed that when providers set expectations for therapy improved interactions: *“She [service provider] told me I would be uncomfortable talking to her at first*. *I told her that I speak about everything sometimes*. *And I want everyone to leave me alone*. *I don’t tell my family what is happening with me*. *She assured me that everything we talk about will be confidential*. *But speaking about your issues will make you relax*, *after a few sessions*. *Honestly*, *I felt comfortable with her*. *She made me feel better”* (male Syrian service user).

### Social environment

MH stigma was reported by both Lebanese and Syrian populations. Participants felt that Syrians are becoming more aware of MH problems and accepting of services, in parallel with attitudes also shifting in Lebanese society. For example, one male PHC manager noted that *“at the beginning of their refuge*, *we didn’t have many Syrians asking about MH*. *They started to be aware about MH after 2017*, *where we started to see Syrians asking about MH*. *They used to have many misconceptions about MH*. *A person may be abused because they have a MH problem*.*”* Several respondents were optimistic that change is happening, albeit slowly, noting that Lebanese populations, in particular men, have become more willing to accept care since the Beirut blast. Additionally, participants felt that more awareness campaigns would help to reduce stigma, especially among with younger generations.

Participants saw stigma and lack of support from partners, family members, friends and community members, impacted care seeking, calling for greater awareness raising. Stigmatisation was noted as a barrier to accessibility, with some individuals fearing societal repercussions or being labelled as *"crazy"* for seeking treatment for MH issues. For example, a female Syrian service user explained: *“First of all*, *people feel ashamed of this issue*, *as if it is a shame if someone is going to a psychologist*, *they think that this person is crazy and abnormal… there is lots of ignorance on this subject*. *So*, *a lot of people hide that they are being treated psychologically or being treated secretly without telling anyone*, *even my husband in the beginning he did not accept anyone to know not at all*.*”* A male Lebanese NGO staff member explained that having MH services delivered within PHCs may reduce stigma related to MH care: *“This is why our [MH] services they’re based in primary healthcare centres*, *so we don’t have a centre for MH alone*, *to avoid stigma and to normalize more the idea that someone is coming to take the service he is taking it as if it’s any other service*, *he is entering a PHC like they see a paediatrician*, *a gynaecologist a dentist*, *they can see also MH services*.*”*

Partners, family members and friends were thus both a barrier and facilitator to accessing MH care. For example, a female Lebanese service user described feeling abandoned and hated by her husband and family due to her symptoms. She spent seven months at her parents’ house and her husband eventually let her return to their home, but she felt unsupported by him and other family members. In other instances, family support, particularly from partners, significantly influenced the decision-making process, emphasising the interconnectedness of MH within familial dynamics. For example, a female Syrian service user explained: *“My husband also he is receiving psychological treatment as me and he encouraged me a lot because he knew how this issue has facilitated things for him and helped him continue his life and to co-exist with his situation that he is living with and how to accept things that he was not accepting before*, *and how he can deal with details that used to make a block for him and stops there*.*”* Interestingly, one participant mentioned how having MH services normalised in the family enables access to MH services: *“My aunt knows I see a psychologist*, *because she sees a psychologist as well*. *So*, *I think she understands this*, *and she gets upset when I don’t take my meds*, *because I get bored of taking meds all the time”* (female Lebanese service user). Participants also described how friends can also play a crucial role in accessing MH services: *“I didn’t know I had a problem*… *but after I got more exposure to the world*, *especially to my friend*, *she told me that I might need to go see someone and this thing is not entirely normal and such*. *And after I started going*, *I started to discover that this is true”* (female Lebanese service user).

For many, the impact of MH issues on their family was also a motivator for seeking support. One female Lebanese service user said that her *"first motive to get treated"* was to be able to be there for her children, as she did not want them to see her in a sick condition. Another female Syrian service user highlighted the role of family in providing motivation for her to get better mentally, saying *"my family is preventing that" when discussing thoughts of ending her life*. *She also expressed a desire to get better to be able to raise her children*: *“That was my hope*, *I was hoping to do something for my family*. *Let’s see the therapy if it will be successful or not*.*”*

### Gender norms

An emerging theme relates to the influence of gender roles and economic control on the ability to afford MH services, even when they are ostensibly free, as out of pocket payments are sometimes required. Participants reported that gender roles and patriarchal systems can affect women–even those who work as they can be denied money for MH services from their husbands. A female PHC manager explained: *“Gender does play a role in how…people seek MH services*, *how they pay for these services*. *Of course*, *we see sometimes households that are headed by men and they don’t see the need for the service*. *It’s then not possible [for their wives] to pay for MH services*.*”*

Another gender dynamic was where women reported finding it challenging to find time to access MH services while juggling work and childcare responsibilities. A female Lebanese service user explained: *“Like a man has*, *like ok*, *his work*, *but the wife has work and children*, *everything she has*.*”* A female Lebanese UN programme manager further noted: *“this is the main difference*, *we see [between men and women]*, *it’s actually sometimes more challenging for woman to actually access MH services because she has children back at home*.*”*

On the other hand, participants also noted the influence of notions of masculinity inhibiting men from discussing MH issues or accessing care. For example, a Syrian father of a child seeking MH services said, *"I am a man and I can handle it*.*"* Participants also described pressure on men to provide for their families, and working long hours, which often prevents them from accessing MH services. Another male Lebanese service user explained: *“In our Eastern community- it is a good thing they established this centre for men only*, *because the community expects that men can’t cry*, *as it is prohibited*, *he can’t express his feeling*. *Whereas females are looked at as more sensitive*. *They would stigmatise men if they have a MH issue*, *unlike women*, *where they consider it normal for her to cry and such*.*”*

### Individual characteristics

Several individual beliefs emerged as barriers to accessing MH services, with reluctance to take medications, harmful coping mechanisms, lack of knowledge and misconceptions of MH therapy, and other concerns related to service users confidence. In contrast, one enabler was identified which is related to service users’ recognising the positive impact of MH therapy, which has been a common theme across the data.

#### Harmful coping mechanisms and seeking alternative MH care

Participants also described how they often turn to harmful coping mechanisms such as drugs and alcohol, which also delayed seeking care. One male Syrian service user participant explained how: *“I was trying to drink to feel like I’m fleeing from the anxiety*, *until I ended having suicidal thoughts due to alcohol and due to anxiety*, *and the alcohol was increasing it*, *I was thinking that alcohol is helping me to flee the anxiety*.*”* Religion was also suggested as an alternative treatment for MH issues by some families, with one female Lebanese service user recounting advice given to her by a sheikh, who did not refer her to further MH services, and simply told her *"that I am feeling like that because of the pregnancy*, *and I wouldn’t feel better until I deliver*.*"*

#### Misconceptions and lack of knowledge about MH issues and the importance of therapy

Participants also had several misconceptions and lack of knowledge on MH problems which pushed them away from seeking MH services. One female Lebanese service user mentioned that her beliefs that her MH state is an illness caused solely by pregnancy, pushed her away from therapy. Other Syrian and Lebanese service users expressed their difficulty differentiating depression and sadness, and low expectations of treatment. One service provider mentioned that service users *“are not able to differentiate that even believers can get suicidal ideation*.*”* Additionally, they described how the widespread belief that MH is not important as discouraging individuals from seeking help. Some service users also expressed fear of misdiagnosis: *“…Especially when you hear before that there were people who were diagnosed wrongly as if they were experimented with*, *so you have fear from such an issue and the fear about your future and where you stand and you really need someone to be next to you”* (female Lebanese service user).

The importance of awareness campaigns was emphasised, with participants calling for more education on MH. One Lebanese service user said: *“I was 19 years old and I stayed 6 years between ups and downs and turning around myself and I didn’t know what I was suffering from until I became 26*, *27 [years of age] and I managed to take the decisions and go [seek MH care]…it is really hard…it took time and I lost years of my life but if I knew [about MH and related services] from the beginning*, *I wouldn’t have lost anything*. *But that is the idea*, *we still need a lot of [MH] awareness or awareness-raising campaigns”* (female Lebanese service user).

#### Concerns related to service users’ own confidence and trust in the MH care process

Several concerns related to service users’ own confidence in therapy have been identified as barriers to seeking support. One is the fear of seeing the therapist in person: *"I would be concerned if I met her face to face*. *She would know my weaknesses*. *I’m afraid to see me as weak or unstable"* (female Lebanese service user). Additionally, some individuals may find it difficult to talk to their therapist, as a female Syrian service user revealed: *"I don’t accept to talk any more about what I am thinking or what is the thing that is making me upset*, *I can’t*, *I can’t*.*"* The fear of being judged or misunderstood also prevented some participants from opening up about MH issues: *"I used to tell myself that I shouldn’t speak*, *not to embarrass myself"* (male Lebanese service user). For some participants, the lack of safety and security to speak openly is a major concern, as a gay male Syrian service user said: *"I was hesitant*…*My whole tribe has threatened to kill me*…*I wouldn’t be able to take care of myself*.*"* Finally, readiness of service users to commence and adhere to therapy can also play a role in seeking MH support, as a NGO manager explained: *"not everyone feels ready to invest into psychological concerns*…*It’s not the priority*, *the priority remains in different areas*, *different goods*, *different services*.*"*

#### Observable positive impact of therapy

Service users consistently expressed the need to seek help and be followed up by service providers and acknowledged the importance of MH services in helping them deal with their problems: *" I don’t know*, *I don’t have the knowledge how to do it*. *So I need to seek help to do it"* (female Lebanese service user). Additionally, participants who saw the benefits of medications were encouraged to continue therapy, with one participant stating *"The most [important] thing I am able to see the effect of is the effect of the medication that is preventing me from entering the vicious cycle*. *This is helping me"* (male Syrian service user). Some participants also viewed their therapist as a helpful friend, which enabled and eased their access to therapy *"I consider her as my friend who is helping me*, *and I should tell her everything"* (female Syrian service user).

### Proposed solutions to improve MH care financing and access

Participants identified several ways to improve financing and access to care (Table 3). It was widely recognised that more national funding is urgently needed for the NMHP and to fully implement the National MH Strategy. Participants also called on the MOPH and NMHP to invest in PHCs and to reduce the pay gap between MH staff employed by MOPH and local and international NGOs, and to broaden the use of task-sharing to include other cadres of non-specialist providers in primary care level to help decrease the current over-reliance on MH specialists.

Proposals related to financing included formulating a comprehensive policy framework for health financing, guaranteeing social health protection to all; urgently raising funds for the NMHP to fully implement the National MH Strategy; exploring innovative financing mechanisms to supplement humanitarian funding; combining social health insurance funds in the NSSF and MOPH funding (from general taxation) in a single-payer pool, and strengthening implementation of the MOPH and NMHP’s commitments towards strategic health financing mechanisms with a focus on PHC by progressively aligning purchasing methods, at least among public purchasers, moving away from passive fee-for-service mechanisms for secondary and tertiary care and including a strong primary care component which should play the role of gate keeper. A full list of proposed solutions is in Table 3.

Proposals related to access care included widespread campaigns that go beyond specific MH conditions such as suicide to include broader aspects of MH. This is seen as crucial in helping individuals recognize and address concerns early on, reducing stigma, and promoting a more supportive environment. Some also called on the MOPH, other relevant governmental ministries, UNHCR, and NGOs to publicise the recently published list of free MH services [37].

A full list of proposals is in Table 3.

## Discussion

To our knowledge, this is the first study to investigate barriers, facilitators, and proposed solutions to MH service financing and access for both Syrian refugees and the Lebanese populations. Our findings highlighted the challenges in financing Lebanon’s system for MH care, including inequitable coverage, dependence on external humanitarian funds, and risks associated with short-term funding and its impact on the sustainability of services for both populations. Our study further revealed how the built environment presents additional challenges to individuals trying to navigate, access and use the available resources, and how the social environment and provider factors enable or hinder individuals from accessing the MH services they need. We found a perception that Syrian refugees registered with UNHCR have better financial coverage to secondary and tertiary MH care as 90% of the fees are covered by UNHCR but, given the economic crisis, both populations are now facing similar challenges in terms of paying for and accessing MH services.

The barriers described are not unique to MH and are found with other chronic conditions [51]. They are exacerbated by the economic crisis and currency devaluation, leading public health institutions to favouring patients who can pay in USD. This has implications for social cohesion, while perceptions of preferential treatment may further exacerbate the social tension between refugee and Lebanese populations [52].

Second, the dependence on external aid and short-term, project-based funding poses a substantial threat to the stability and sustainability of MH programmes. This threat necessitates the establishment of a more robust, long-term financing model [53], which has also been recommended in Lebanon’s national health strategy [54], and underpins the imperative for external donors to support and strengthen the national system rather than investing in parallel systems. Moreover, our findings highlight the need for the MOPH to formulate a comprehensive framework for health financing guaranteeing social health protection to all, and in parallel, work on how to reduce fragmentation of the health financing system. It is also critical that MOPH implement its commitments to combining social health insurance funds in the NSSF and MOPH funding into a single-payer pool, in order to maximise efficiency and increase the scope for cross-subsidies to vulnerable populations. This is especially important given that as of March 2023, 80% of Lebanese are reported to be living below the relative poverty line, including around 36% below the extreme poverty line [18], and in 2022, 90% of Syrian families needed support to meet their basic survival needs [16]. Going beyond commitments, it is important that this work on reforming the health financing system is done a timely fashion, with accountability mechanisms put in place to monitor progress. However, the stalemate to find a president and make steps towards resolving the current political and economic crisis is a barrier to the government finding sustainable solutions for a number of issues, including that of a better financed health sector.

In addition to the challenges, we also identified important enablers for a sustainable MH care system including the efforts of MOPH and NMHP integrating MH into PHCs. The Primary Health Care Unit successfully defined a set of the essential healthcare services, with the aim to make them financially accessible through subsidised packages for all residents [55]. This plan has contributed positively to greater equity between Lebanese populations and Syrian refugees at the primary care level, where everyone can access the same services [55]. Ongoing efforts of the MOPH and NMHP in integrating MH services into more sustainable care models such as the primary care network, though heavily dependent on external aid, would enhance the overall MHPSS response, as has been shown in other humanitarian settings where integration increased communities’ access to these services [56].

Our participants suggested the need for MOPH to secure dedicated funding to the NMHP given its strategic oversight and leadership in scaling up the MH response in the country. Funding a national structure dedicated to MH, like the NMHP, can also help bridge the gap between the priorities of policymakers and practitioners on the ground, and bring together other sectors working on MHPSS, to coordinate and amplify impact. This recommendation also comes in line with the current MHPSS guidelines [4, 33]. Despite the efforts of the NMHP in integrating MH into PHCs and incorporating refugee responses into national strategies, these efforts will be ineffective if the system is not well-resourced [57, 58]. For instance, studies have shown that reaching sustainable efforts necessitates endorsement from authorities, encompassing the national government to institutionalise MH care nationwide. This entails the establishment of legal and policy frameworks enabling long-term funding and facilitating the intervention’s growth through the development and the commitment to national guidelines and strategic policy documents [59]. In Lebanon’s case, this would entail finalising and more importantly, securing funding for its National MH Strategy 2023–2030 [38].

The shortage of MH specialists and brain drain, and reported lack of compassion from some service providers, emphasise the significant gaps within the healthcare environment which needs to be addressed for more equitable and quality public MH care [60]. The need for retention of workforce solutions needs to be addressed. These barriers associated with the health system suggests that for an equitable MH care, these context specific characteristics need to be considered in the national efforts [61]. For instance, the large gap between the demand for MH services and the limited number of MH professionals signals the need for MOPH and MHPSS partners to continue working on implementing and scaling up innovative and efficient approaches to tackle this problem, which include task shifting and incentivising the recruitment and training of MH care providers [62–66]. Our study reported some service users reporting frustration and perceived lack of compassion from MH providers, emphasising the need for better quality care which should be addressed via consistent training packages for all MH providers, with monitoring of tracer quality of care indicators for MH at national level [60, 67]. Our study also reported service users noting that MH services are not currently being provided in a way that meet their preferences or understanding of MH challenges, which underscore the importance of providing culturally relevant MH services tailored to both Syrian and Lebanese populations.

Our study also found demand-side barriers to accessing MH care including stigma, lack of support from family, friends and partners, and lack of relevance of MH services, for example not having a choice of gender of service provider when seeking services, as well as services not being offered in a group format. These findings are consistent with recent reviews focused on barriers to seeking care in low-and middle-income countries [68], and among refugee [69] and child and adolescent populations [70]. Addressing these barriers requires comprehensive and inclusive mental health policies and legislations that lead to improvements in MH services, as well as sustainable and culturally adapted MH awareness programmes, including in communities, schools and work settings, that are co-created with target populations.

Our study has several limitations which should be addressed with future research. The self-reported nature of data collected in the key informant interviews and FGDs is a limitation. The sensitive nature of the study topic may have also created reporting bias in the interviews and FGDs, with some service providers, governmental stakeholders, NGO and UN staff not being able to speak freely in fear of making showcasing their services or agencies in a negative light. Despite trying to sample participants from all relevant stakeholder groups, we were unable to interview any participants from NSSF or the MOPH department that is responsible for paying for health services as the “last resort” funder. Additionally, although we aimed to collect data from equal numbers of male and female service users, we found it more challenging to sample males. This led to an uneven number of males and female service users being recruited, which may have led to gender bias in our study results. Finally, this study focused only focused on the health system, while we are aware that MHPSS is an intersectoral issue and responsibility. Future research should assess the provision of MH care through other sectors, such as protection, livelihoods, and education.

## Conclusions

Multiple crises in Lebanon have further exacerbated challenges in health system financing for MH care, dependence on external humanitarian funds, and risks and sustainability issues associated with short-term funding. Urgent reforms are needed to Lebanon’s health financing system to equitably and efficiently finance and scale up MH care with a focus on PHCs [54], and to reduce inequities in MH service coverage between Lebanese and Syrian refugee populations. In particular, external donors should consider channelling humanitarian funding into the national system, rather than into a parallel system. Moreover, there is an urgent need for the government to raise funds for the NMHP and to fully implement the National MH Strategy [38], while also working on wider health financing reforms by: strengthening implementation of the MOPH’s commitments towards strategic health financing mechanisms with a focus on PHC; combining social health insurance funds in the NSSF and MoPH funding into a single-payer pool; and reducing fragmentation at all levels by strengthening NSSF and fostering a national consensus on how to cover the entire population Lebanon in an equitable way with no or little contributory capacity.

## Supporting information

S1 Checklist Inclusivity in global research. (DOCX)
